# No means ‘No’: a non-improper modeling approach, with embedded speculative context

**DOI:** 10.1093/bioinformatics/btac593

**Published:** 2022-08-30

**Authors:** Priya Tiwary, Akshayraj Madhubalan, Amit Gautam

**Affiliations:** Saama AI Research Lab, Pune 411057, India; Saama AI Research Lab, Pune 411057, India; Saama AI Research Lab, Pune 411057, India

## Abstract

**Motivation:**

The medical data are complex in nature as terms that appear in records usually appear in different contexts. Through this article, we investigate various bio model’s embeddings (BioBERT, BioELECTRA and PubMedBERT) on their understanding of ‘negation and speculation context’ wherein we found that these models were unable to differentiate ‘negated context’ versus ‘non-negated context’. To measure the understanding of models, we used cosine similarity scores of negated sentence embeddings versus non-negated sentence embeddings pairs. For improving these models, we introduce a generic super tuning approach to enhance the embeddings on ‘negation and speculation context’ by utilizing a synthesized dataset.

**Results:**

After super-tuning the models, we can see that the model’s embeddings are now understanding negative and speculative contexts much better. Furthermore, we fine-tuned the super-tuned models on various tasks and we found that the model has outperformed the previous models and achieved state-of-the-art on negation, speculation cue and scope detection tasks on BioScope abstracts and Sherlock dataset. We also confirmed that our approach had a very minimal trade-off in the performance of the model in other tasks like natural language inference after super-tuning.

**Availability and implementation:**

The source code, data and the models are available at: https://github.com/comprehend/engg-ai-research/tree/uncertainty-super-tuning.

## 1 Introduction

Most of the medical data like discharge summaries, pathology reports and radiology reports are in textual form, these data are then used for various research and clinical analysis purposes. Nowadays, artificial intelligence (AI) has been proven as one of the prominent sources for conducting such experiments and deriving insights from these clinical data. Detecting negation, speculation in such sensitive records is one of the unavoidable prerequisites in many information retrievals, extraction tasks and building intelligent systems such as a system providing decisive criteria for recruiting a patient or not in cohort selection for any clinical trial. Hence, it is really important to have models that understand the context of terms appearing in the medical records data.

At present, we have various state-of-the-art (SOTA) models in the biomedical domain [BioBERT (Lee *et al.*, 2019), BioELECTRA (Kanakarajan *et al.*, 2021) and PubMedBert (Gu *et al.*, 2020)] which have performed great in various bio NLP tasks. We investigated these models’ capabilities of understanding negative context by calculating the cosine distance between non-negative sentences (e.g. NAC had an effect on the half-life of E-selectin or VCAM-1mRNA) and their corresponding negative sentence (e.g. NAC had **no** effect on the half-life of E-selectin or VCAM-1mRNA) pairs. We found that none of these models were able to differentiate between the sentence pairs as the cosine distances between them were very less and approximately close to 1. This experiment gave us insight into the model’s inability to understand negations.

To enhance these models’ embeddings for helping them understand the negation and speculation context in medical data, we propose a unique methodology of super-tuning the models using a proposed synthesized dataset. To create the synthesized dataset, we start with negation medical sentences from the BioScope full-text corpus (excluding abstracts) which is a negation cue annotated dataset by building a parser class to extract the negative sentences and their corresponding cue. Later, we transform these negative sentences into their affirmative sentences manually. Then to increase the data points, we used paraphraser models like T5 (Raffel *et al.*, 2020), Pegasus (Zhang *et al.*, 2019) and generated new sentence pairs. In the end, we generated 56667 sentence pairs data points and a corresponding score was assigned to it in a range of −1 to 1.

We now super-tune the model on these 5.6k data points with Cosine Similarity Loss with a Siamese network structure. For each sentence pair, we pass sentence A and sentence B through our network which yields semantically meaningful embeddings and can be compared with cosine similarity. This process is known as super-tuning and this allows our network to recognize if negation and/or speculation is present in sentences. We now fine-tune this super-tuned model on different tasks like detecting cue (e.g. This is not a lump. ‘not’ being predicted as cue) and scope (e.g. This is not a lump. ‘a lump’ being predicted as scope) in a sentence. The major contributions of our work can be summarized as follows:

- Created and published a synthesized dataset that can be utilized for making any bio model understand the negation context.
- Introduced a super tuning method that can be a plugin before the fine-tuning task to make the embeddings smarter in identifying negation and the speculation context in a sentence.
- The resultant model achieved SOTA on negation, speculation cue and scope detection tasks on BioScope abstracts as well as on the Sherlock dataset.

## 2 Literature survey

To date, all the well-known algorithms and models in the negation and speculation area have been focused on detecting cues and their scope in sentences. These algorithms have been developed using various intuitive approaches such as rule based, statistical machine learning and deep learning.

Chapman *et al.* (2001) developed NegEx, a rule-based approach that makes use of regular expressions and was designed for determining whether a disease is present or absent in a medical diagnosis report. Another rule-based approach model NegFinder was developed by Mutalik *et al.* (2001) which is a lexical scanner that generates a finite state machine and a parser built on regular expressions. The team from U Washington, White *et al*. (2012) applied their rule-based idea of using regular expressions on the Sherlock dataset and scored an F1 score of 90 on the dataset. Apart from these, Peng *et al.* (2017) developed NegBio, a rule-based approach that utilizes patterns on universal dependencies to identify the scope of triggers that are indicative of negation or speculation, and achieved an F1 score of 95.9 on the BioScope dataset.

One disadvantage of rule-based approaches is that they do not generalize well for different domain data as well as need customization of rules for new corpus or domain. This problem does not occur while using machine learning algorithms. In 2009, Morante and Daelemans (2009) used a memory-based learning algorithm (IGTREE), for detecting cues. For scope resolution, they used three classifiers (memory-based learning algorithm, SVM and CRF) which predicted whether a given word is the beginning of a scope, end of the scope or neither. It achieved SOTA on BioScope abstracts and full papers negation cue detection task by gaining F1 scores of 98.68 and 97.81, respectively. Other negation models using machine learning were developed by Agarwal and Yu (2010) and Councill *et al.* (2010) utilizing popular statistical approaches such as support vector machines (SVM) and conditional random fields (CRF), respectively.

**Fig. 1.**
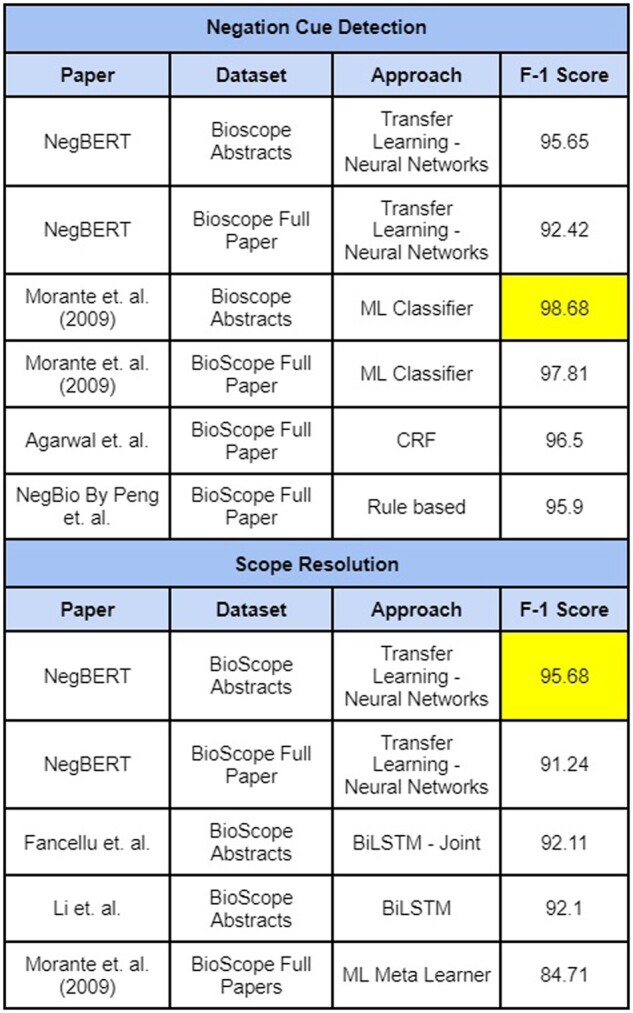
Performance of existing models on BioScope cue, scope detection

**Fig. 2.**
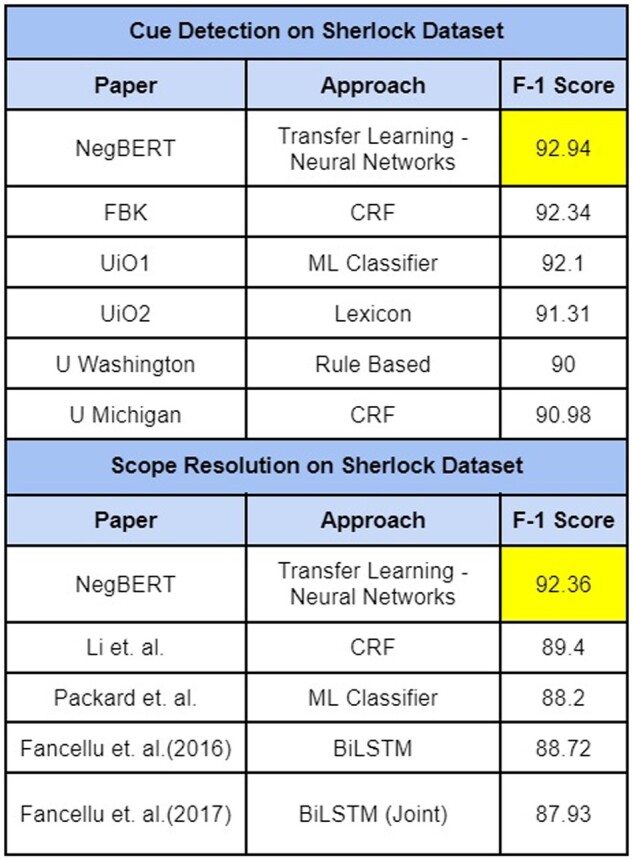
Performance of existing models on Sherlock cue, scope detection

Various neural network approach-based modeling has been developed in negation scope detection tasks. Qian *et al.* (2016) developed a convolutional neural network (CNN) based architecture that first classifies whether a token is a negation cue or not and later uses a CRF layer at the last layer to output a sequence determining the scope of negation in the input sentence. Fancellu *et al.* (2017) developed a negation cue detection model wherein a dependency tree is passed as input to LSTM architecture. Chen (2019) used attention-based deep learning architecture to detect negation and assertion in clinical notes. Transfer-learning approach was used by Khandelwal and Sawant (2020) wherein they trained BERT (Vaswani *et al.*, 2017) model on BioScope, Sherlock and SFU datasets. Their resultant model achieved SOTA on Sherlock Cue, Scope detection, BioScope Abstracts, and Full papers Scope Resolution and SFU Scope Resolution tasks. Models with an average 85 F1-score on detecting cue, scope from BioScope and Sherlock dataset through different approaches have been depicted in Figures 1 and 2.

## 3 Methodology

### 3.1 Super-tuning dataset preparation

The BioScope corpus(Szarvas *et al.*, 2008) includes three different sub-corpora: Abstracts of biological papers from the GENIA corpus (Collier *et al.*, 1999), full scientific papers from Flybase and BMC Bioinformatics website, and clinical radiology records corpus. Its medical and biological texts have been annotated for negation and their linguistic scope. This was done to allow a comparison between the development of systems for negation/hedge detection and scope resolution. We build a custom parser that filters out the negation data from only the Bioscope’s full scientific papers excluding abstracts. Along with this, we manually created a dataset with negation and their corresponding affirmation. Thus, as a result, we form sentence pairs which we then assign three scores (−1,0,1). Score −1 indicates that the cosine similarity between the sentences in the pair should be more as though the sentences are giving information regarding the same thing but contextually are opposite. Score 0 indicates that the two sentences in a pair are completely different in terms of context hence the cosine similarity should be 0. Score 1 indicates that the sentences in a pair are similar in context hence cosine similarity between them should be 1.

Once we have the above data i.e. sentence pairs with their labels we pass them to paraphrase models: T5 by Raffel *et al.* (2020) and Pegasus by Zhang *et al.* (2019). Paraphrasing is a task that creates new sentences for an input sentence that expresses the same meaning using a different choice of words. T5 paraphraser is an encoder-decoder structured transformer pretrained on 750 GB of diverse texts which uses Google’s Universal Sentence Encoder (USE) (Cer *et al.*, 2018) to create an embedding of each sentence. These embeddings are 512-D vectors that are produced in such a way that related sentences will be closer to each other in the vector space than unrelated sentences. The Pegasus paraphraser is also a transformer model which was pretrained in a similar way of summarization wherein important sentences are removed and masked from the document for the model to recover them in the output. We pass each sentence pair of all the three scores (−1,0,1) to output new four sentence pairs with the same meaning as the input sentence pair but with different choices of words on all the three labels. With this method, out of 15 296 sentence pairs altogether of the three scores (−1,0,1), we were able to generate 56 999 new sentence pairs.

### 3.2 Super-tuning strategy

**Fig. 3.**
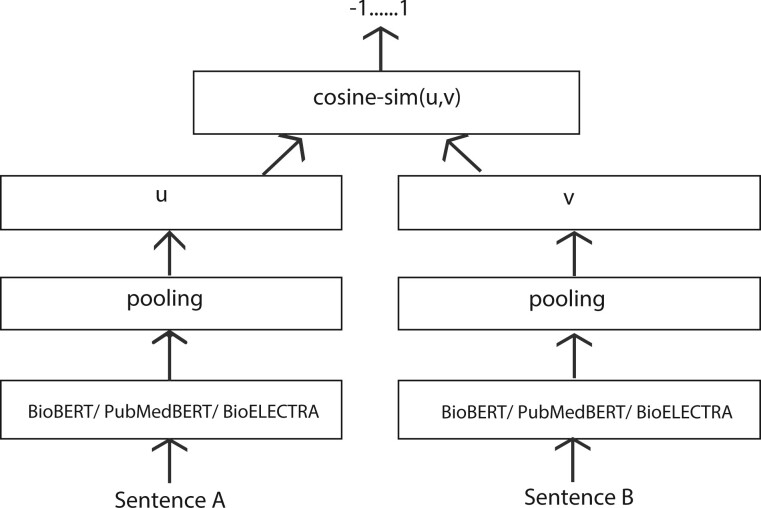
Super-tuning architecture

The dataset that we developed was of sentence pairs with their respective scores (−1,0,1) which indicates cosine similarity between the sentences of a pair. We used the architecture of sentence-BERT implemented by Reimers and Gurevych (2019) which used cosine similarity loss for training. It is the Siamese and triplet network that updates the weights such that the produced sentence embeddings are meaningful semantically and then can be compared with cosine-similarity. The model adds a pooling operation to the embedding input from the existing model as shown in Figure 3.

The cosine similarity loss function takes two sentence embeddings (*u, v*) and returns their similarity score between −1 and 1 as depicted in Figure 3. Equation 1 states the formula for the calculation of cosine similarity loss between embedding spaces *u, v*.

(1) $$\mathrm{similarity}=\text{cos}(\theta )=\frac{u\cdot v}{\left||u||\;||v|\right|}=\frac{\sum_{i=1}^{n}u_{i}v_{i}}{\sqrt{\sum_{i=1}^{n}u_{i}^{2}}\sqrt{\sum_{i=1}^{n}v_{i}^{2}}}$$

The mean squared error loss is used on the original label versus the predicted cosine similarity as shown in equation 2, where *Y_i_* represents the given cosine similarity score for the embedding pair *u, v*. The model uses the loss function, updates its weights and tries to minimize the loss.

(2) $$\mathrm{MSE}=\frac{1}{n}\sum_{i=1}^{n}(\text{cos}(\theta )-Y_{i})$$

We incorporated different bio-model embeddings such as BioBERT, PubMedBERT and BioELECTRA by replacing BERT at the input embedding layer with these three model’s embeddings at every round of the experiment as shown in Figure 3. BioBERT, PubMedBERT and BioELECTRA are pre-trained language models for the biomedical domain. The BioBERT model has been pre-trained on Pubmed data for 1M steps. The PubMedBERT has also been pre-trained on Pubmed data, but it differs from BioBERT in the initialization of weights. BioBERT is initialized using BERT weights which have been pre-trained on the general domain corpora whereas PubMedBERT has been pre-trained from scratch and just on pure domain-specific data. Both these models are based on BERT architecture which is a transformer-based model using a multilayer and multi-head self-attention mechanism. The BioELECTRA model is based on ELECTRA architecture which comprises a generator and discriminator. It has also been trained on Pubmed data from scratch. The results of incorporating these three models in the described SBERT architecture which we name as NegBioBERT, NegPubMedBERT and NegBioELECTRA are stated in Section 4.1.

### 3.3 Fine-tuning tasks

We checked all our three models NegBioBERT, NegPubMedBERT and NegBioELECTRA on two tasks: natural language inference (MedNLI) and sentence similarity (BIOSSES). MedNLI (Shivade, 2019) dataset consists of sentence pairs developed by Physicians annotated for definitely true, maybe true and definitely false. The dataset contains 11 232 training, 1395 development and 1422 test instances. BIOSSES (Soğancıoğlu *et al.*, 2017) is a benchmark dataset for biomedical sentence similarity estimation. The dataset comprises 100 sentence pairs, in which each sentence was selected from the Text Analysis Conference (TAC) Biomedical Summarization Track Training Dataset containing articles from the biomedical domain. The sentence pairs were evaluated by five different human experts that judged their similarity and gave scores in a range [0–4]. As per our initial results on the super-tuning approach, we found NegBioELECTRA outshining the other two models hence, we used it for Negation, Speculation cue, and scope detection on Bioscope abstracts and Sherlock datasets respectively. We also ensure that there is no data leakage as none of the data in super tuning are re-used in any fine-tuning tasks. Since Bioscope’s full papers are used for super-tuning we used only abstracts for fine tuning. For negation, speculation cue and scope detection on the BioScope and the Sherlock (Mirsky, 2016) datasets, we use a similar transfer learning approach as NegBERT introduced by Khandelwal and Sawant (2020). Our approach differs from NegBERT in deciding the labels for the cue detection model. Unlike NegBERT, we have provided separate classes for negation, and speculation cues. So, our model is capable of finding if the detected cue is negative or speculative. We provide the following labels in our token classification approach to each word/sub-word for the BioScope dataset:

*0 - Padding*

*1 - Normal word*

*2 - Negation Cue*

*3 - Speculation Cue*

And for the Sherlock dataset the labels are as follows:

*0 - Padding*

*1 - Normal word*

*2 - Cue*

We output negation, speculation sentences and their cues, scopes using our built parser as mentioned in Section 3.1. We label each token in every sentence using the above-mentioned labels and create the dataset to be fed to the cue detection model which is illustrated as per the BioScope dataset in one example as:

**Input Sentence:** This may not be a lesion

**Label per token:** [1,3,2,1,1,1]

We use our NegBioELECTRA model’s tokenizers for tokenization as well as add padding tokens so that the length of input and output matches. These data are then passed to the model which provides probabilities of label per token. We do the required post-processing to get one label per token or word of the sentence. The whole flow of the cue detection model is depicted in the following example:

**Input Sentence:** This may not be a lump

**Label per word:** [1,3,2,1,1,1]

**Tokenized i/p:** [[CLS],This,may,not,be,a,lump,[SEP][PAD],[PAD],.]

**Label per token:** [1,1,3,2,1,1,1,1,0,0,0,0.]

**Model’s output:** [[0.02, 0.95, 0.02, 0.01], [0.01, 0.95, 0.01, 0.03], [0.00, 0.04, 0.00, 0.96], [0.01, 0.03, 0.95, 0.01], [0.00, 0.96, 0.02, 0.02], [0.02, 0.95, 0.02, 0.01], [0.02, 0.96, 0.02, 0.01], [0.02, 0.97, 0.02, 0.00], [0.00, 0.98, 0.01, 0.01], x, x.]

**Post-processed op:** [1,3,2,1,1,1]

For scope resolution, we use binary labels i.e. 1 and 0 to label per token of a sentence if it is a word in scope or not, respectively. Also, we annotate the negation, speculation cues in a sentence by adding [NEG] and [SPE] before and after the cue, respectively. These two are our special tokens with the help of which we are trying to make understand the model that cues are present in the sentence and their location. If there are multiple cues in a sentence, then we send sentences annotating them per cue per instance manner. Illustration to understand this process as follows:

**Input Sentence Instance 1:** This [SPE] may [SPE] not be a lump

**Label per word:** [0,0,1,1,1,1]

**Input Sentence Instance 2:** This may [NEG] not [NEG] be a lump

**Label per word:** [0,0,0,1,1,1]

**Fig. 4.**
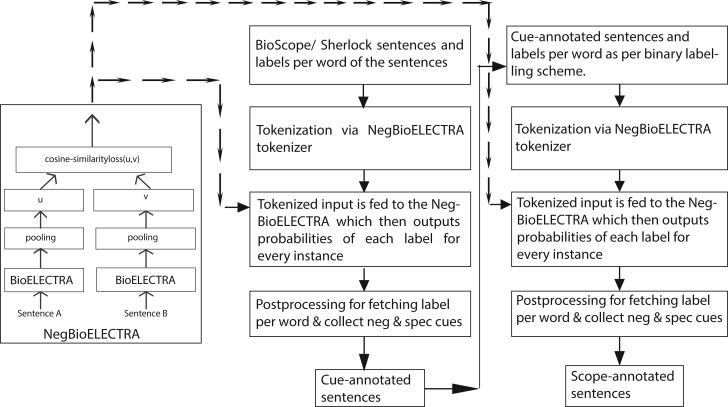
A descriptive diagram illustrating the flow of super-tuning and using the model for negation, speculation cue, and scope detection tasks on the BioScope and the Sherlock datasets. The super-tuned model acts as the base model for both cue and scope detection tasks

After data preparation, we use a similar method as cue detection wherein we use our NegBioELECTRA model’s tokenizer for tokenization as well as add padding tokens so that the length of input and output matches. These data are then passed to the model which provides probabilities of label per token. We do the required post-processing to get one label per token or word of the sentence. The whole architecture is proposed in Figure 4.

## 4 Experiments and results

### 4.1 Experimental setup

**Fig. 5.**
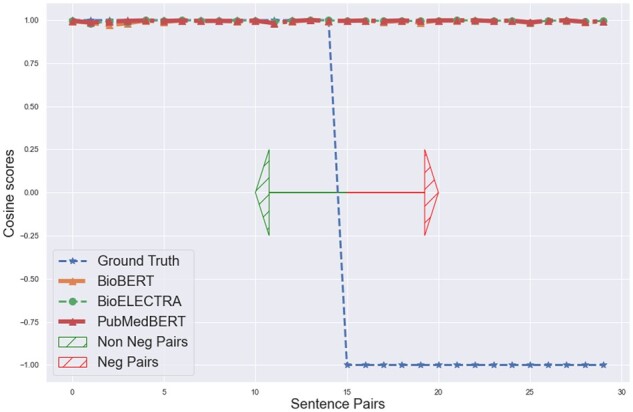
Original BioModels Cosine scores

We evaluated different bio-model embeddings such as BioBERT, PubMedBERT and BioELECTRA on their understanding of negation context. For this evaluation, we took some sentence pairs among which one sentence is negative and the other is its affirmed sentence. Then output their embeddings from each of the three models and evaluate the cosine similarity between them. We found that the cosine similarity of each of the sentence pairs from all these models was approximately 0.99 (depicted in Fig. 5) which stated that the model embeddings failed to understand the negation context.

Now, we replace the input embedding layer in the SBERT architecture with BioBERT, PubMedBERT and BioELECTRA embedding layer one by one as depicted in Figure 4. We feed our prepared training data as explained in Section 3.1 and train each model with a different embedding layer for 10 epochs. Once each of these models is super-tuned we check which resultant model has performed well in terms of cosine similarity score. The models are fine-tuned on the MedNLI and BIOSSES dataset on different learning rates [1e^−5^, 1.5e^−4^, 2e^−5^, 2.5e^−4^, 3e^−5^, 5e^−5^], batch sizes [16,32] and epochs [5–30] to check if our approach had any effect on the performance of the original models. The best model is fine-tuned on the negation and speculation cue, scope detection task on the BioScope abstracts and Sherlock datasets as described in Section 3.3 on different learning rates [1e^−5^, 1.5e^−4^, 2e^−5^, 2.5e^−4^, 3e^−5^, 4e^−5^, 5e^−5^], batch sizes [16,32] and epochs [5–30]. We ensured that the train, test and validation data splits were made according to previous SOTA achieved model’s standards.

### 4.2 Results

**Fig. 6.**
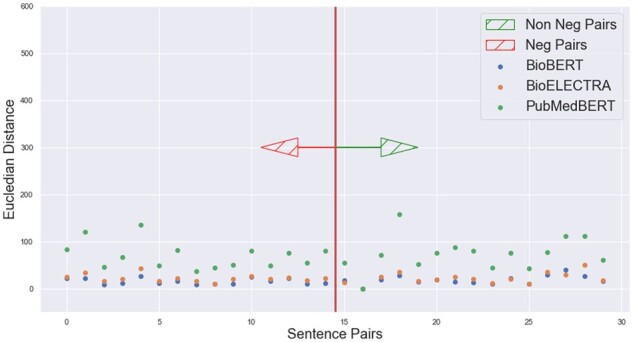
Original BioModels Euclidean distances

**Fig. 7.**
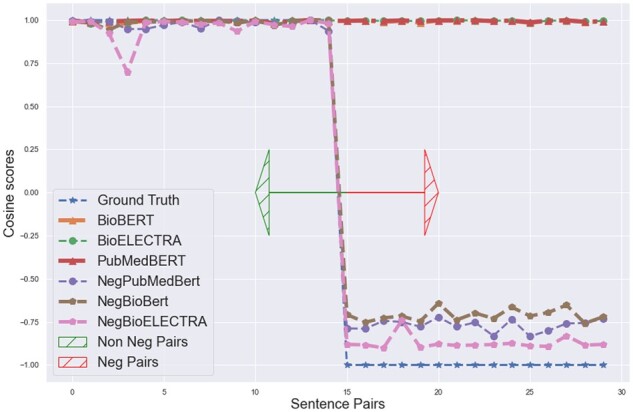
Original versus super-tuned BioModels Cosine scores

**Fig. 8.**
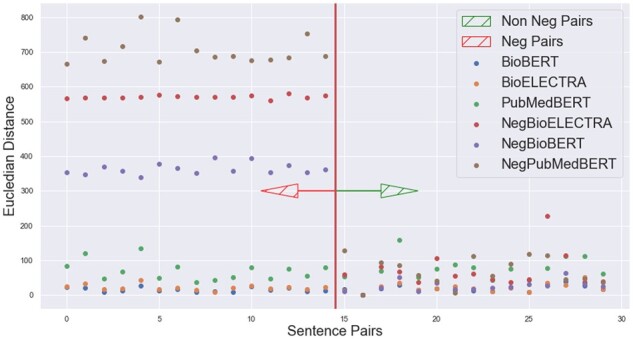
Original versus super-tuned BioModels Euclidean distances

We retrieve the new sentence embedding from each of the models which we name as NegBioBERT, NegPubmedBERT, NegBioELECTRA for some of the negation and its affirmation sentence pairs. We then calculate the cosine similarities between each sentence pair embeddings output from the three models and compare them. We found that our super-tuning approach has successfully made these embeddings understand the negation context as now the distance between the embedding of each sentence pair from all three models is more and not approximately equal to 0.99 as it was earlier depicted in Figure 5. This comparison can be clearly seen in Figure 7 where we plot the sentence pairs tagged as data points on the X-axis and cosine similarity on the Y-axis. Similar improvement can be seen in terms of Euclidean distance in Figure 8 by comparing with results in Fig. 6 where we plot the sentence pairs tagged as data points on the X-axis and the Euclidean distance between each pair on the Y-axis. We also found that the super-tuning approach had a minimal trade-off in accuracy with respect to the original model on test data of the MedNLI and BIOSSES dataset.

**Fig. 9.**
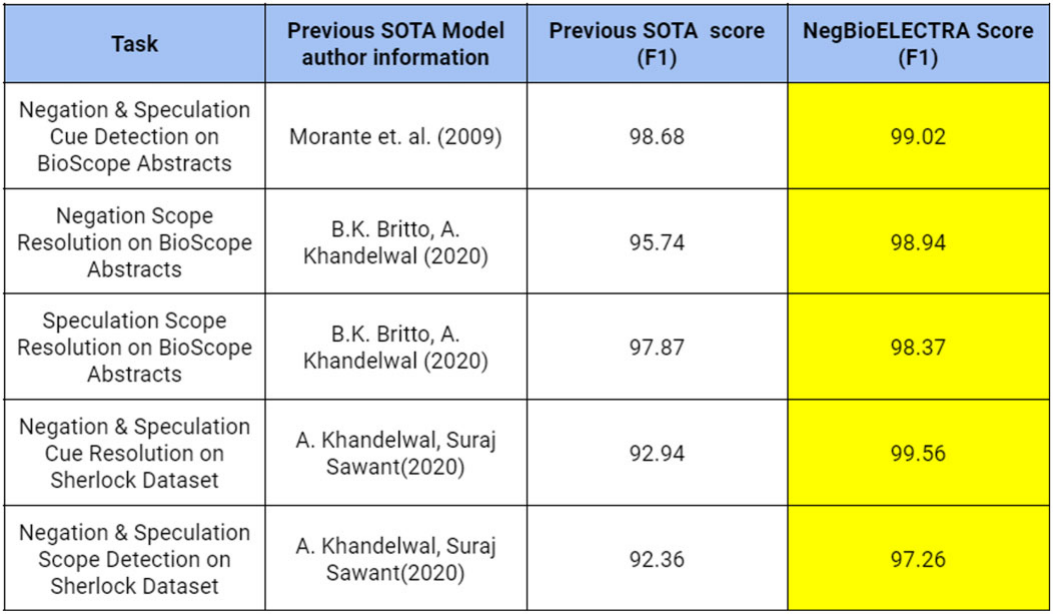
Previous state-of-the-art versus NegBioELECTRA scores

We used NegBioELECTRA for negation, speculation cue and scope detection tasks on BioScope, Sherlock datasets. On the test data, we found NegBioELECTRA out-performing all the current models in the respective task. On all the tasks mentioned in Figure 9, we observed a mean gain of 1.35. For negation, speculation cue detection on BioScope Abstracts, we achieved an F1 score of 99.02 thus beating the previous SOTA score by 0.34 points. For Negation Scope Resolution on BiosScope Abstracts, we found our model outperformed by scoring an F1 score of 98.94 and beating the previous SOTA by 3.2 points. Along with the other two tasks, the model also gained 0.5 points in the earlier SOTA score in Speculation Scope Resolution on BioScope Abstracts by scoring an F1 score of 98.37. The model outperformed and marked the same progress in the Sherlock dataset cue detection task by scoring an F1 score of 99.56 and beating the previous SOTA by 6 points. Similar progress was seen in the Sherlock Scope Resolution task wherein the model gained 4.9 points over the previous SOTA by scoring an F1 score of 97.26.

## 5 Conclusion

We release the super-tuned BioELECTRA i.e. the NegBioELECTRA model which for any given sentence predicts negation or speculation cue and then generates scope for that particular cue. For carrying out the described super-tuning approach, we synthetically produced a dataset using T5 and Pegasus paraphraser on a manually curated BioScope dataset. Our results show that this super-tuning approach has enriched the existing model’s capabilities in negation, speculation cue and scope detection as well as its embeddings understanding on the negation, speculation context. We have achieved SOTA on the following tasks: BioScope abstracts negation, speculation cue as well as scope detection, Sherlock’s negation cue and scope detection. NegBioELECTRA unlike its predecessors has the ability to detect and identify the cue as negative or speculative. The great results of super-tuned bio models working on general domain datasets are because the same cues are present in the domain and non-domain datasets. The cues that the models have been trained on for the respective tasks hence are limited. Hence, we feel more datasets with new cues are needed to bring more revolutions in the negation and speculation area of AI. Along with this, we encourage research to focus more on curating domain-specific as well as general-domain datasets for the super-tuning approach. This will help the models to be aware of the speculation in every domain. We feel that every emerging natural language model should have the ability to understand negated and speculative sentences. Thus, we need more such super-tuning approaches which enhance the model’s capabilities of understanding the negation and speculation context with a minimal trade-off on its performance on other tasks. A sentence cannot only be negative or speculative but also declarative, exclamatory, imperative, interrogative, and sarcastic or opinionated. Hence, our interest greatly relies upon developing a generic model which can understand all of these forms of sentences.

## References

[btac593-B1] Abu-Jbara A. , Radev D. (2012) A conditional random field model for resolving the scope of negation. In: **SEM 2012: The First Joint Conference on Lexical and Computational Semantics – Volume 1: Proceedings of the main conference and the shared task, and Volume 2: Proceedings of the Sixth International Workshop on Semantic Evaluation (SemEval 2012)*, *Montréal, Canada*. Association for Computational Linguistics, pp. 328–334.

[btac593-B2] Agarwal S. , Yu H. (2010) Biomedical negation scope detection with conditional random fields. J. Am. Med. Inform. Assoc., 17, 696–701. 20962133 10.1136/jamia.2010.003228 PMC3000754

[btac593-B3] Britto B.K. and Khandelwal A. (2020) Resolving the scope of speculation and negation using transformer-based architectures. ArXiv:abs/2001.02885.

[btac593-B4] Cer D. et al (2018) Universal sentence encoder. In: *Proceedings of the 2018 Conference on Empirical Methods in Natural Language Processing: System Demonstrations*, Brussels, Belgium. Association for Computational Linguistics, pp. 169–174.

[btac593-B5] Chapman W. et al (2001) A simple algorithm for identifying negated findings and diseases in discharge summaries. J. Biomed. Inform., 34, 301–310. 12123149 10.1006/jbin.2001.1029

[btac593-B6] Chen L. (2019) Attention-based deep learning system for negation and assertion detection in clinical notes. IJAIA, 10, 1–9.

[btac593-B7] Chowdhury M.F.M. (2012) FBK: exploiting phrasal and contextual clues for negation scope detection. In: **SEM 2012: The First Joint Conference on Lexical and Computational Semantics – Volume 1: Proceedings of the main conference and the shared task, and Volume 2: Proceedings of the Sixth International Workshop on Semantic Evaluation (SemEval 2012)*, *Montréal, Canada*. Association for Computational Linguistics, pp. 340–346.

[btac593-B8] Clark K. et al (2020) ELECTRA: pre-training text encoders as discriminators rather than generators. In: *8th International Conference on Learning Representations, ICLR 2020, Addis Ababa, Ethiopia, April 26-30, 2020*.

[btac593-B9] Collier N. et al (1999) The GENIA project: corpus-based knowledge acquisition and information extraction from genome research papers. In: *Ninth Conference of the European Chapter of the Association for Computational Linguistics,Bergen, Norway. *Association for Computational Linguistics, pp. 271–272.

[btac593-B10] Councill I. et al (2010) What’s great and what’s not: learning to classify the scope of negation for improved sentiment analysis. In: *Proceedings of the Workshop on Negation and Speculation in Natural Language Processing, Uppsala, Sweden. *University of Antwerp, pp. 51–59.

[btac593-B11] Devlin J. et al (2018) BERT: pre-training of deep bidirectional transformers for language understanding. *Association for Computational Linguistics, *N19-1423, 4171-4186. https://doi.org/10.18653/v1/N19-1423.

[btac593-B12] Fancellu F. et al (2016) Neural networks for negation scope detection. In: *Proceedings of the 54th Annual Meeting of the Association for Computational Linguistics (Volume 1: Long Papers)*, *Berlin, Germany*. Association for Computational Linguistics, pp. 495–504.

[btac593-B13] Fancellu F. et al (2017) Detecting negation scope is easy, except when it isn’t.In: *Proceedings of the 15th Conference of the European Chapter of the Association for Computational Linguistics: Volume 2, Short Papers*, *Valencia, Spain*. Association for Computational Linguistics, pp 58–63.

[btac593-B14] Fancellu F. et al (2018) Neural networks for cross-lingual negation scope detection.*arXiv:1810.02156v1* Clin. Orthop. Relat. Res. (CoRR).

[btac593-B15] Gu,Y. et al (2022) Domain-specific language model pretraining for biomedical natural language processing. ACM Trans. Comput. Healthcare, 3, 1–23. 10.1145/3458754.

[btac593-B16] Kanakarajan K.R. et al (2021) BioELECTRA: pretrained biomedical text encoder using discriminators. In: *Proceedings of the 20th Workshop on Biomedical Language Processing*, Online. Association for Computational Linguistics, pp. 143–154.

[btac593-B17] Khandelwal A. , Sawant S. (2020) NegBERT: a transfer learning approach for negation detection and scope resolution. In: *Proceedings of the 12th Language Resources and Evaluation Conference*, *Marseille, France*. European Language Resources Association, pp. 5739–5748.

[btac593-B18] Lapponi E. et al (2012) Sequence-labeling negation using dependency features, SEM 2012. In: **SEM 2012: The First Joint Conference on Lexical and Computational Semantics – Volume 1: Proceedings of the main conference and the shared task, and Volume 2: Proceedings of the Sixth International Workshop on Semantic Evaluation (SemEval 2012)*, *Montréal, Canada*. Association for Computational Linguistics, pp. 319–327.

[btac593-B19] Lee J. et al (2019) BioBERT: a pre-trained biomedical language representation model for biomedical text mining. Bioinformatics, 1367–4803. 10.1093/bioinformatics/btz682 PMC7703786 31501885

[btac593-B20] Li H. , Lu W. (2018) Learning with structured representations for negation scope extraction. In: *Proceedings of the 56th Annual Meeting of the Association for Computational Linguistics (Volume 2: Short Papers)*, *Melbourne, Australia*. Association for Computational Linguistics, pp. 533–539.

[btac593-B21] Mirsky Y. (2016) SherLock vs moriarty: a smartphone dataset for cybersecurity research. In: *Proceedings of the 2016 ACM Workshop on Artificial Intelligence and Security (AISec '16)*. Association for Computing Machinery, New York, NY, USA, pp. 1–12.

[btac593-B22] Morante R. , Daelemans W. (2009) A metalearning approach to processing the scope of negation. In: *Proceedings of the Thirteenth Conference on Computational Natural Language Learning (CoNLL-2009)*, *Boulder, Colorado*. Association for Computational Linguistics, pp. 21–29.

[btac593-B23] Mutalik P. et al (2001) Use of general-purpose negation detection to augment concept indexing of medical documents: a quantitative study using the UMLS. J. Am. Med. Inform. Assoc., 8, 598–609. 11687566 10.1136/jamia.2001.0080598 PMC130070

[btac593-B24] Packard W. et al (2014) Simple negation scope resolution through deep parsing: a semantic solution to a semantic problem. In: *Proceedings of the 52nd Annual Meeting of the Association for Computational Linguistics (Volume 1: Long Papers)*, *Baltimore, Maryland*. Association for Computational Linguistics, pp. 69–78.

[btac593-B25] Peng Y. et al (2017) Negbio: a high-performance tool for negation and uncertainty detection in radiology reports. AMIA Jt Summits Transl Sci Proc., 188–196. PMC5961822 29888070

[btac593-B26] Qian Z. et al (2016) Speculation and negation scope detection via convolutional neural networks. 10.18653/v1/D16-1078.

[btac593-B27] Raffel C. et al (2020) Exploring the limits of transfer learning with a unified text-to-text transformer. J. Mach. Learn. Res., 21, 1–67.34305477

[btac593-B28] Read J. et al (2012) UiO1: constituent-based discriminative ranking for negation resolution. In: **SEM 2012: The First Joint Conference on Lexical and Computational Semantics – Volume 1: Proceedings of the main conference and the shared task, and Volume 2: Proceedings of the Sixth International Workshop on Semantic Evaluation (SemEval 2012)*, *Montréal, Canada*. Association for Computational Linguistics, pp. 310–318.

[btac593-B29] Reimers N. , Gurevych I. (2019) Sentence-BERT: sentence embeddings using Siamese BERT-networks. In: *Proceedings of the 2019 Conference on Empirical Methods in Natural Language Processing and the 9th International Joint Conference on Natural Language Processing (EMNLP-IJCNLP)*, *Hong Kong, China*. Association for Computational Linguistics, pp. 3982–3992.

[btac593-B30] Shivade C et al. (2019) MedNLI - a natural language inference dataset for the clinical domain. In: *Proceedings of the 2018 Conference on Empirical Methods in Natural Language Processing*, *Brussels, Belgium*. Association for Computational Linguistics. pp. 1586–1596.

[btac593-B31] Soğancıoğlu G. et al (2017) BIOSSES: a semantic sentence similarity estimation system for the biomedical domain. Bioinformatics, 33, i49–i58. 28881973 10.1093/bioinformatics/btx238 PMC5870675

[btac593-B32] Szarvas G. et al (2008) The BioScope corpus: annotation for negation, uncertainty and their scope in biomedical texts. In: Proceedings of the Workshop on Current Trends in Biomedical Natural Language Processing, Columbus, Ohio. Association for Computational Linguistics, pp. 38–35.

[btac593-B33] Vaswani A. et al (2017) Attention is all you need. In: *Proceedings of the 31st International Conference on Neural Information Processing Systems (NIPS'17)*. Curran Associates Inc., Red Hook, NY, USA, pp. 6000–6010.

[btac593-B34] White J.P. (2012) UWashington: negation resolution using machine learning methods, SEM 2012. In: *The First Joint Conference on Lexical and Computational Semantics – Volume 1: Proceedings of the Main Conference and the Shared Task, and Volume 2: Proceedings of the Sixth International Workshop on Semantic Evaluation (SemEval 2012)*. pp. 335–339.

[btac593-B35] Zhang J. et al (2019) PEGASUS: pre-training with extracted gap-sentences for abstractive summarization. *In: Proceedings of the 37th International Conference on Machine Learning (ICML'20). JMLR.org, Article 1051*, pp. 11328–11339.

